# Makes FLASH the difference between the intervention group and the treatment-as-usual group in an evaluation study of a structured education and treatment programme for flash glucose monitoring devices in people with diabetes on intensive insulin therapy: study protocol for a randomised controlled trial

**DOI:** 10.1186/s13063-018-2479-9

**Published:** 2018-02-05

**Authors:** Melanie Schipfer, Carmen Albrecht, Dominic Ehrmann, Thomas Haak, Bernd Kulzer, Norbert Hermanns

**Affiliations:** 1 Research Institute Diabetes Academy Bad Mergentheim (FIDAM GmbH), Johann-Hammer-Str. 24, 97980 Bad Mergentheim, Germany; 2 Diabetes Center Bad Mergentheim, Theodor-Klotzbücherstr. 12, 97980 Bad Mergentheim, Germany

**Keywords:** Diabetes, Flash glucose monitoring, Patient education and treatment programme, Psychoeducation, Self-management, Patient-reported outcome

## Abstract

**Background:**

People with diabetes on intensive insulin therapy need sufficient glycaemic control to prevent the onset or progression of diabetic complications. The burden of multiple daily blood glucose self-testing can be lessened by novel diabetes technology like flash glucose monitoring systems which provide more information compared to self-monitoring of blood glucose. Despite this delivered additional information studies are showing no significant effect on HbA_1c_ reduction, but a reduced time spent in a hypoglycaemic glucose range. We assume that users of these devices need additional education and training to integrate the delivered information into treatment decisions. Therefore, FLASH, an education and treatment programme, was developed. The programme evaluation follows herein.

**Methods/design:**

Patients are recruited through 40 diabetes outpatient study centres located across Germany. They will be randomly assigned to participate in the education and treatment programme (intervention group) or to obtain treatment as usual (control group). All patients have to give blood samples and to answer a bench of questionnaires during baseline assessment, at the end of the intervention, and 6 months after the end of the intervention. Physicians will be asked to declare some additional clinical data (such as details of the diabetes therapy) for every patient at every one of the three assessment points.

**Discussion:**

This study is conducted as a randomised controlled trial to test the hypothesis that the newly developed education and treatment programme combined with the use of a flash glucose monitoring device (intervention group) is superior to reduce HbA_1c_ compared to the use of flash glucose monitoring alone (control group). The first results will be expected in 2018.

**Trial registration:**

ClinicalTrials.gov, ID: NCT03175315. Registered on 2 May 2017.

**Electronic supplementary material:**

The online version of this article (10.1186/s13063-018-2479-9) contains supplementary material, which is available to authorized users.

## Background

Diabetic patients on intensive insulin therapy with multiple daily injections or continuous, subcutaneous insulin infusion need to achieve sufficient glycaemic control to prevent the onset or progression of diabetic complications [1, 2], while at the same time avoiding the risk of severe hypoglycaemia.

Successful intensive insulin treatment requires close self-monitoring of blood glucose (SMBG). Measurement of blood glucose is especially important before main meals because the prandial insulin dose will vary with blood glucose level, planned carbohydrate consumption, and other factors (e.g. exercise, alcohol consumption). The multiple daily skin pricks and the need to carry a blood glucose meter, test strips, lancets, and blood glucose log books can be very trying, and patients often get frustrated [3]. However, novel technologies, such as flash glucose monitoring, can provide relief.

Flash glucose monitoring provides continuous measurement of interstitial glucose levels via a glucose sensor placed in the subcutaneous tissue of the upper arm [4, 5]. This sensor can be left at the insertion site for 14 days, and the interstitial glucose level obtained whenever necessary by scanning with a reader or smartphone using near-field technology. In addition to the current glucose level, the reader or smartphone will also display arrows indicating the long-term trend in interstitial glucose level (slightly/strongly increasing, slightly/strongly decreasing, or stable) and the course of the glucose level over the last 8 h.

Flash glucose monitoring has several benefits over SMBG. Multiple daily skin pricks can be avoided. Glucose levels can be painlessly measured any number of times by scanning with a reader and, moreover, the results are automatically stored for 90 days. Software can display glucose patterns and provide information on additional parameters, such as time spent in predefined glycaemic ranges (e.g. hypoglycaemic or hyperglycaemic range), median and mean glucose values, or an estimation of the current glycated haemoglobin level (HbA_1c_), based on the previous collected and stored interstitial glucose values.

This new technology also has some downsides. Users need to be educated on how to integrate all the additional information (e.g. trend arrows, previous course of glucose) into treatment decisions. Problematic glucose patterns requiring treatment adjustments can be easily identified, but only if the user has the necessary knowledge and skills.

Two previous studies [1, 2] have shown that flash glucose monitoring has no significant effect on HbA_1c_, although it does reduce time spent in the hypoglycaemic glucose range. We hypothesise that education on how to make optimal use of flash glucose monitoring devices would improve outcomes. We therefore developed a programme, FLASH, to support these patients. We are planning a randomised controlled trial (RCT) to determine the effect of FLASH on HbA_1c_ as well as on secondary outcomes (i.e. time spent in different glucose ranges, patient-reported outcomes, and device satisfaction). The purpose of this paper is to present the research protocol of the planned study.

## Methods/design

### Study setting

The study is designed as a multicentre study. A total of 40 diabetes outpatient treatment centres located across Germany have been invited to recruit eligible patients for the study. A study centre is defined as a medical practice run by a diabetologist and where diabetes nurses or certificated diabetes educators (CDE) are also available. The course instructors for FLASH will be CDE. Prior to the study, the course instructors will undergo an intensive 8-h training in the study protocol and the education and treatment programme. This programme will be conducted by the research team. Course instructors will be provided with a written curriculum and audiovisual teaching material, along with detailed descriptions of each slide. Additionally, trainers from the coordination research institute (Research Institute of Diabetes Mergentheim: FIDAM) will visit each participating medical centre, and course instructors can then resolve any doubts about the conduct of the programme. In these visits FIDAM employees will confirm that the slides of the FLASH will operate and could be displayed in each medical praxis and that the course instructor knows how to conduct the programme, fully understands the content of the programme, can clarify any likely questions, and knows how to upload the therapy data from the flash glucose monitoring reader to Diasend®. During the course of study, all study centres will have a hotline to FIDAM for support. FIDAM will communicate with all participating study centres via newsletters where every new development can be presented promptly and reliably. Conversely, the coordinating centre will be available for the responsible personnel in the study centres for the entire duration of the study. In addition to conducting the education and treatment programme, the physicians at the study centres will be responsible for the therapy of their patients during and after the study.

### Study design

This multicentre RCT will enroll diabetic patients on intensive insulin therapy who are using flash glucose monitoring. The patients will be randomised into one of two groups: an intervention group and a control group. Both groups will receive the flash monitoring device and technical instructions on its use; the intervention group will receive, in addition, FLASH training.

### Sample size calculation

A total of 86 patients per group will be necessary to detect a mean difference of 0.3% and a standard deviation of 0.7% in HbA_1c_ reduction between the two treatment groups (Cohen’s *d* = 0.43), with an alpha error = 0.05 and beta = 0.2 (power = 0.8). Given an expected non-evaluable rate of 20%, a total of 216 participants will be needed, with 108 patients in each group.

### Recruitment and randomisation

All participating study centres will identify eligible patients interested in participating in the study. The eligible patients will be offered an appointment to get to know the facts of the study, to clarify doubts, and to give signed informed consent. Because no education programme for patients using flash glucose monitoring devices is available, medical centres and patients are showing great interest in participating in this study. However, despite the great interest it is possible that the required sample size will not be achieved. In this case there are more medical centres available which can be contacted for patient recruitment. Patient recruitment will be stopped after 216 patients are enrolled.

Baseline assessment will take place over two visits. At the first visit, blood will be collected, baseline questionnaires will be completed, patients will be provided with the flash glucose monitoring device and receive technical instructions, and the sensor will be implanted. Blinded FIDAM staff will review all baseline assessments to confirm that the enrolled patients meet all the eligibility criteria. Then, randomisation of patients will be done centrally at FIDAM using SYSTAT version 12, with random assignment at the level of the study centre. The results will be sent to the respective study centres in sealed envelopes that will be opened only at the second baseline visit. At this second visit, 2 weeks after the first visit, patients will be informed of the group assignment. Glucose-level data from the reader will be uploaded via Diasend® (see below: ‘Assessments’).

### Inclusion and exclusion criteria

#### Inclusion criteria

Patients of either sex will be eligible for inclusion in the study if they fulfil the following conditions:

- Age 16–75 years
- On intensified insulin therapy/insulin pump therapy
- Previous participation in at least one structured diabetes education programme
- HbA_1c_ in the range of 7.5–14%
- Reduction of HbA_1c_ as the therapeutic goal
- Able to understand, speak, and write German
- Willing to provide informed consent (if necessary, informed consent of the parents)
- Have an indication for using a flash glucose monitoring system

Indication for a flash glucose monitoring system will be decided by the treating physician. The decision will be based on the following: (1) frequent unexplicable glucose levels and need for multiple daily measurement of blood glucose; (2) severe hypoglycaemic events, especially during the night; hypoglycaemic unawareness; and (3) undue patient anxiety about the use of the lancet, but otherwise good compliance with the device.

#### Exclusion criteria

Patients will be excluded if they have any of the following:

- Diabetes duration < 1 year
- Type 2 diabetes not on insulin, or on non-intensified insulin therapy
- Severe organic disease preventing regular participation in the training course
- Pregnancy
- Severe cognitive impairment
- Current treatment of a psychiatric disorder
- Renal disease requiring dialysis

The treating physician will confirm that each patient satisfies the inclusion and exclusion criteria by checking the health records or by clinical assessment. Once included in the study, patients may still be excluded from the analysis if they decide to leave the study at any point for any reason, or if they miss any one treatment appointment or one of the three assessment points.

The REPLACE and IMPACT studies [1, 2] were not able to show significant improvement in glycaemic control with flash glucose monitoring vs. SMBG. Assuming that education might be able to improve the efficacy of flash glucose monitoring, exclusion of patients with previous flash glucose monitoring system use is not necessary (since the device alone does not seem to be efficacious in improving the HbA_1c_). The main objective of this study is not to prove the efficacy of flash glucose monitoring systems, but rather to evaluate the efficacy of the FLASH education and treatment programme. However, if there are substantial differences between the control and intervention groups in previous flash glucose monitoring system use, adjustment will be made during the analysis for experience of using previous flash glucose monitoring systems.

### Content, structure and intervention of FLASH

FLASH is a structured education and treatment programme that will be delivered in a group setting, with each group comprising three to eight patients in the age range 16–75 years. This wide age range was selected so that study centres could include as many eligible patients as possible. Previous experience has shown that it is difficult to recruit homogeneous age groups. Having patients of widely differing ages is not necessarily a disadvantage, since younger people with diabetes can learn from the experiences of older ones and vice versa. If important differences are observed between the different age groups, adjustment for these factors will be made during analysis of data.

FLASH aims to empower patients by providing information and teaching strategies for better self-management of diabetes. FLASH participants are encouraged to be actively involved in their treatment: they formulate their own therapy-related goals, learn to recognise patterns in their glucose levels and to identify trends, and also to use this information for optimising daily therapy decisions. During FLASH, participants are also invited to share their experiences with flash glucose monitoring with other group members. The ultimate objective is to teach patients to live active and normal lives through efficient use of flash glucose monitoring.

FLASH uses different modern educational techniques. The guiding principle is self-management, especially self-monitoring, self-assessment, and enhancing self-treatment. Participants are provided with written material and worksheets and are encouraged to test the contents of each lesson on their own and to then discuss their experiences in the group setting. They are introduced to computer-based data analysis software and learn how to use it for optimising their own therapy. Additionally, patients define what they personally hope to achieve by attending the FLASH programme. Course instructors are provided with core content about the interpretation of flash glucose monitoring results and also optional content in slides on a secondary level if more detailed information is needed on a certain topic (e.g. exercising or hypoglycaemia).

The FLASH programme takes place over 6 weeks. It consists of four sessions, lasting 90 min each. There is a 1-week interval between sessions 1 and 2, and 2-week intervals between sessions 2 and 3 and sessions 3 and 4. These intervals give patients time to practise the newly learned techniques in their daily lives. Table 1 provides an overview of FLASH.

**Table 1:** Overview of FLASH: aims, content, and didactics

Session	Aim	Key content	Special didactical feature
1 First week	Information about, and motivation for, using of flash glucose monitoring	Principles of flash glucose monitoring	Clarification of features of the device
		Understanding trend arrows	Personal motivation
2 Second week	Recognition of glucose pattern	Analysing glucose values and trends	Introduction to the 6 modules on AGP: steps 1–2: understanding the AGP.
			Intensive discussion about glucose value documentation and computer-based data analysis software
3 Fourth week	Therapy adjustment based on pattern recognition and AGP	Using data to recognise glucose patterns and to adjust the therapy	The 6 modules (continued): modules 3–6: interpreting the AGP, and therapy adjustment.
			Intensive discussion and amplification by personal examples
4 Sixth week	Check of the adjustment of the therapy	Dealing with barriers	Reinforcement of lessons learned
			Preparing methods for long-term management

*AGP* ambulatory glucose profile

The *first session* focusses on the function of the flash glucose monitoring system and on interpretation of the trend arrows. The emphasis will be on teaching patients how to make therapy adjustments based on the information derived from the trend arrows.

The *second session* introduces patients to ambulatory glucose profile (AGP) monitoring (modules 1–2 of the six modules on AGP). Patients will learn to systematically analyse their glucose profiles and to organise the information from the flash glucose monitoring system.

The *third session* focusses on pattern recognition (modules 3–6). Participants will learn how to interpret the AGP and use it for therapy adjustment. Simple case studies will be used to teach participants to recognise typical patterns. Possible causes for these patterns and the appropriate therapy adjustments will be discussed.

The *fourth* and final session focusses on the changes in the AGP due to therapy adjustments. Individual cases on therapy adjustment during FLASH will also be discussed. Participants will be given an opportunity to share practical tips on how to handle minor day-to-day problems.

FLASH was developed in consultation with a group of experts (diabetologists and diabetes educators with special experience in flash glucose monitoring) according to German and international guidelines. The contents and lessons were discussed with the experts over the course of 1 year and were refined based on patients’ experiences with flash glucose monitoring. FLASH has a written curriculum to be followed by educators and study material for patients like worksheets used during courses as well as tasks for the time at home in which the patients will have to test the content of the preceding course.

**Fig. 1:**
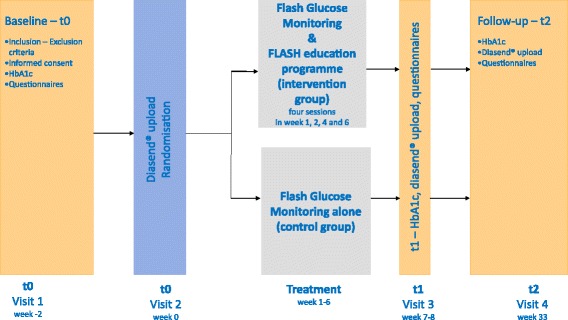
The evaluation of FLASH – study flowchart

Figure 1 shows how the patients were assigned to the two treatment arms.

### Outcome measures

#### Primary outcome

The primary outcome will be change in glycaemic control analysed by estimating HbA_1c_.Testing will be performed in a central laboratory using the high-performance liquid chromatography (HPLC) method (normal range, 4.3–6.1% or 23.5–43.2 mmol/mol). Laboratory personnel will be blinded to the treatment allocation. All blood samples will be analysed once and then destroyed; specimens will not be stored for future use.

#### Secondary outcomes

Secondary outcomes will include medical and psychosocial measurements. These secondary outcomes will include: (1) time spent in different glycaemic ranges (hypo-/normo-/hyper-glycaemic status: < 70 mg/dL/70–180 mg/dL/> 180 mg/dL); variability of glucose level; (2) episodes of severe hypoglycaemia; (3) quality of life; (4) diabetes distress; (5) depression; (6) empowerment; and (7) satisfaction with the device and with the education.

### Assessments

Patients will be assessed at *three time points* (Fig. 1):

1. At baseline (t0)
2. At the end of FLASH, after the fourth session (t1)
3. At follow-up 6 months after the end of FLASH (t2)

The first assessment will take place, as described above, over two visits with an interval of 2 weeks. The second assessment will occur immediately at the end of the FLASH programme or within 2 weeks after its end. At this visit the patients’ glycaemic status and satisfaction with the device will be assessed. Additionally, in the intervention group, satisfaction with the FLASH programme will also be evaluated. The third and last assessment will be at follow-up 6 months after the end of the FLASH programme. At this visit patients will answer follow-up questionnaires, give blood samples, and return the flash glucose monitoring device to the study personnel.

All assessments will be made by the personnel at the respective centre. All study centres are instructed by a personal visit and each study centre was offered a hotline and was audited by telephone. In addition, 50% of the study centres will be audited by FIDAM to check the quality of study conduct. Glycaemic data from the flash glucose monitoring system will be uploaded via Diasend®; thus, FIDAM can identify online incomplete data and notify the study centres whenever necessary.

Glucose values recorded through the flash glucose monitoring reader will be uploaded through the web-based diabetes management system Diasend®, which will be installed on computers at each study centre. With individual patient codes, all flash glucose monitoring data will be uploaded by the responsible personnel at each study centre during the visits. Demographic data (age, gender, nationality, family status, housing situation, and educational level) and additional clinical data (weight and height, comorbidities, and details of the diabetes therapy) will be entered into Case Report Forms (CRFs) by the concerned physician. All CRFs will be in paper-and-pencil form; no electronic Case Report Forms (eCRFs) will be used. FIDAM will be responsible for the management of all collected data. This study is registered at ClinicalTrials.gov (identifier: NCT03175315) where all relevant information is available to the public. There will be no external safety committee since this is a non-pharmacological intervention. The incidence of serious adverse events and device-related adverse events will be collected via standardised reporting forms and reported to FIDAM.

Data on participants’ emotional state and their experiences in living with diabetes will be collected through diabetes-, programme-, and device-related questionnaires:

- Diabetes Acceptance Scale (DAS) – to assess illness acceptance. Patients mark their intensity of agreement with 28 statements on a 4-point scale from 0 (not true) to 3 (totally true). A higher score indicates a higher level of acceptance [6]
- Diabetes Distress Scale (DDS) – a validated self-report scale to assess the current level of diabetes-related emotional distress. It has 28 items rated on a 6-point scale from 0 (no problem at all) to 5 (a very serious problem) [7]
- Problem Areas in Diabetes (PAID) Scale – a self-report form which builds up an index of emotional pressure, using 20 items rated on a 5-point scale from 0 (not a problem) to 4 (serious problem) [8]
- General quality of life – the widely used World Health Organisation Well-being Index (WHO-5) self-report form to assess general quality of life. The scale consists of five questions, rated on a 6-point scale from 0 (at no time) to 5 (all the time). It is a measure of sense of well-being over the last 2 weeks [9]
- Health-oriented Quality of Life (EQ-5D; German version) – to assess the actual health status and to grade problems in five fields [10]
- Centre for Epidemiologic Studies Depression Scale (CES-D; German version) – to assess depression; it uses nine items rated on a 4-point scale from 0 (never/rarely) to 3 (often/always) [11]
- Personal Health Questionnaire (PHQ-8) – to assess mood issues over the last 2 weeks; eight items are rated on a scale that ranges from 0 (never) to 3 (nearly every day) [12]
- Hypoglycaemia Fear Survey (HFS) – to assess worries and behaviour related to hypoglycaemic events; it uses 18 statements related to troubles with hypoglycaemia at a variety of events; patients rate the troubles experienced on a 5-point scale that ranges from 0 (never) to 4 (always) [13]
- Diabetes Empowerment Scale – to assess empowerment and psychosocial self-efficacy; it uses 21 items to examine how difficulties in the daily routine are handled [14]
- Hypoglycaemia Awareness Scale (German version) – this consists of nine items. Scores range from 0 (maximum hypoglycaemic awareness) to 9 (minimum hypoglycaemic awareness) [15]
- Satisfaction with diabetes therapy – patients answer 10 questions on how satisfied they were with several aspects of their diabetes therapy over the last 4 weeks, with higher score indicating a higher level of dissatisfaction [16]
- Satisfaction with FLASH – the intervention group will be asked to evaluate FLASH. Participants will answer a total of 16 items
- Fidelity measure – in addition as a fidelity measure an 11-item scale will be applied. Participants of FLASH will be asked to what degree the educators addressed individual goals, how supported they felt in adjusting their diabetes therapy, and if the programme was presented in a structured way
- Glucose Monitoring Satisfaction Survey (GMSS) – patients will be asked to indicate their thoughts and emotions while using the flash glucose measurement system; there are 15 items that can be scored on a scale ranging from 1 (does not apply at all) to 5 (applies very strongly) [17]
- Use of technical possibilities – patients will be asked to describe their use of all the technical possibilities of the flash glucose monitoring system within the last 4 weeks; responses are graded on a scale ranging from 0 (not at all) to 4 (several times a day)
- Experiences with glucose measurement (Glucose Monitoring Assessment Tool; GMAT) – 19 statements are used to build up a score that reflects experiences related to measurement of glucose level; for example, ‘the measurement of glucose is awkward for me’ or ‘I avoid measuring my glucose level if I have the feeling that the level is high’. [18]

**Fig. 2:**
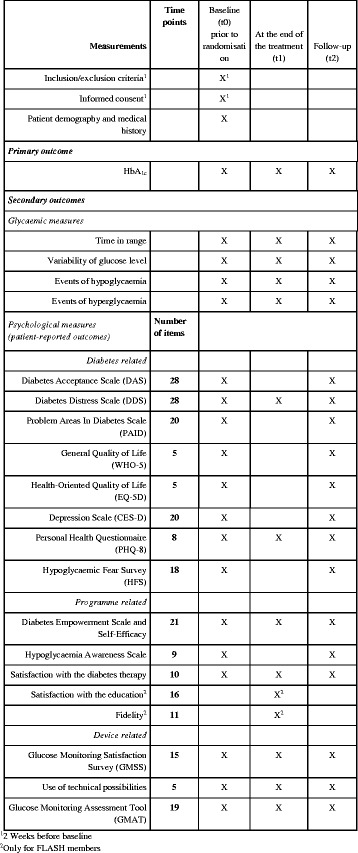
Overview of all measurements at the different assessment points

Answering all the questionnaires will take about 30 min for each patient (the booster questionnaire administered at the end of the FLASH programme is slightly smaller). Figure 2 presents a summary of all the measurements.

Financial grants will be issued for patients who complete the study. These financial grants will not be given to the patients but to the study centres to compensate them for the additional effort (i.e. recruitment of patients, informing the patients about the study, obtaining informed consent, conduct of treatment, checking questionnaire data and electronic data for completeness, answering queries, and reporting serious or device-related adverse events). All educational materials will be supplied free to the study centres and patients.

### Planned data analysis

Continuous data over all three time points will be summarised using descriptive statistics. Differences across the time points will be calculated for each group. The distributions of these differences will be compared using analysis of variance (ANOVA) for repeated measurements, with the FLASH group as the independent variable. If significant baseline differences are seen, the respective baseline values will be used as covariates.

Sometimes the questionnaire data may be skewed and may not fulfil the criteria for interval data. Assuming that at least rank-order data are available from the questionnaires, we will use either non-parametric tests for the evaluation of questionnaires and glucose data, or perform a transformation of the scores and glucose parameters into van der Waerden scores before applying parametric tests. Based on the data distribution and the scale of measurement, appropriate statistical methods will be selected. In the case of categorical data determined over several time points, we will use logistic regression analysis with adjustment for baseline values.

Statistical testing will be conducted at a significance level of *α* = 0.05. All analyses will be performed using SYSTAT version 12.0. For the intention-to-treat analysis missing data will be substituted by the last-observation-carried-forward method.

All data will be treated as confidential and be stored at FIDAM, the study coordination centre, for 10 years. Only FIDAM personnel will have access to the data during the study. FIDAM will monitor the flow of participants through each stage of the trial, and finally will be able to publish it.

## Discussion

For quality of reporting, all standard protocol items recommended for international trials were taken in consideration according to the SPIRIT (Standard Protocol Items: Recommendations for Interventional Trials) Checklist, providing recommendations for a minimum set of ethical, scientific, and administrative elements which should be addressed in a clinical protocol [19] (see Additional file 1).

Patient enrolment will take place during the summer break and vacation periods and may be at different times at different centres.

The main results will be presented in conferences and reported in peer-reviewed publications. If FLASH is found to be beneficial the programme will be certified in Germany, so at the end every concerned patient will benefit.

### Trial status

Protocol version number: 02.01.2018_1 (4 January 2018).

The study is currently (at the time of submission) recruiting participants. This can be seen at ClinicalTrials.gov (NCT03175315). Patient recruitment started on 2 May 2017, and will be completed by early October 2017. The first results are expected to be available in 2018.

## Additional file

Additional file 1: SPIRIT 2013 Checklist: recommended items to address in a clinical trial protocol and related documents*. (PDF 168 kb)

## Abbreviations

AGP: Ambulatory glucose profile
CDE: Certificated diabetes educators
CES-D: Centre for Epidemiologic Studies Depression Scale
CRF: Case Report Form
DAS: Diabetes Acceptance Scale
DDS: Diabetes Distress Scale
eCRF: Electronic Case Report Form
EQ-5D: Health-oriented Quality of Life – German version
FIDAM: Research Institute of Diabetes Mergentheim
FLASH: Name of the new patient education and treatment programme
GMAT: Glucose Monitoring Assessment Tool
GMSS: Glucose Monitoring Satisfaction Survey
HbA_1c_: Glycated haemoglobin A
HFS: Hypoglycaemia Fear Survey
HPLC: High-performance liquid chromatography
PAID: Problem Areas In Diabetes
PHQ-8: Personal Health Questionnaire
RCT: Randomised controlled trail
SMBG: Self-monitoring blood glucose
SPIRIT: Standard Protocol Items: Recommendations for Interventional Trials
SYSTAT: Statistical software, San Jose, CA, USA
WHO-5: World Health Organisation Well-being Index
